# Comparative Analysis of Impact Strength among Various Polymeric Materials for Orthotic Production

**DOI:** 10.3390/polym16131843

**Published:** 2024-06-28

**Authors:** Rachel Habiba, Ana Amaro, Daniela Trindade, Carla Moura, Rui Silva, André Antão, Rui F. Martins, Cândida Malça, Ricardo Branco

**Affiliations:** 1 Department of Mechanical Engineering, University of Coimbra, Rua Luis Reis Santos, 3030-788 Coimbra, Portugal; 2 Center for Rapid and Sustainable Product Development (CDRSP), Polytechnic of Leiria, 2430-028 Marinha Grande, Portugal; daniela.trindade@ipleiria.pt (D.T.); carla.moura@ipc.pt (C.M.); rui.d.silva@ipleiria.pt (R.S.); andre.a.costa@ipleiria.com (A.A.); candida@isec.pt (C.M.); 3 CEMMPRE-ARISE, Department of Mechanical Engineering, University of Coimbra, Rua Luis Reis Santos, 3030-788 Coimbra, Portugal; ana.amaro@dem.uc.pt; 4 Applied Research Institute, Polytechnic Institute of Coimbra, Rua da Misericórdia, Lagar dos Cortiços, S. Martinho do Bispo, 3045-093 Coimbra, Portugal; 5 Abel Salazar Biomedical Sciences Institute (ICBAS), University of Porto (UP), Rua de Jorge Viterbo Ferreira, No. 228, 4050-313 Porto, Portugal; 6 Research Center for Natural Resources Environment and Society (CERNAS), Polytechnic Institute of Coimbra, Bencanta, 3045-601 Coimbra, Portugal; 7 CIPER, Faculdade de Motricidade Humana, Universidade de Lisboa, 1495 Cruz Quebrada Dafundo, 1649-004 Lisbon, Portugal; 8 UNIDEMI, Department of Mechanical and Industrial Engineering, Nova School of Science and Technology, Universidade NOVA de Lisboa, Campus de Caparica, 2829-516 Caparica, Portugal; rfspm@fct.unl.pt; 9 Coimbra Institute of Engineering (ISEC), Polytechnic Institute of Coimbra, Rua Pedro Nunes, Quinta da Nora, 3030-199 Coimbra, Portugal

**Keywords:** additive manufacturing, biomedical applications, impact strength, orthotics, polymeric material, sweat solution

## Abstract

Orthotic devices play an important role in medical treatment, addressing various pathologies and promoting patient recovery. Customization of orthoses to fit individual patient morphologies and needs is essential for optimal functionality and patient comfort. The advent of additive manufacturing has revolutionized the biomedical field, offering advantages such as cost reduction, increased personalization, and enhanced dimensional adaptability for orthotics manufacturing. This research focuses on the impact strength of nine polymeric materials printed by additive manufacturing, including an evaluation of the materials’ performance under varying conditions comprising different printing directions (vertical and horizontal) and exposure to artificial sweat for different durations (0 days, 24 days, and 189 days). The results showed that Nylon 12 is good for short-term (24 days) immersion, with absorbed energies of 78 J and 64 J for the vertical and horizontal directions, whereas Polycarbonate (PC) is good for long-term immersion (189 days), with absorbed energies of 66 J and 78 J for the vertical and horizontal directions. Overall, the findings contribute to a better understanding of the suitability of these materials for biomedical applications, considering both short-term and long-term exposure to physiological and environmental conditions.

## 1. Introduction

Orthoses are medical devices prescribed to (i) correct different pathologies; (ii) limit, redirect, or impose specific movements or forces; (iii) minimize potential injuries; (iv) promote injury recovery; or (v) correct anomalies, congenital or not [1]. Regardless of the objective, given how it works, adapting the orthosis—i.e., customization—to the patient’s morphology is crucial. Customized orthoses are widely recognized as being advantageous, both for their functionality in medical terms, allowing greater efficiency in immobilization or restriction of movement, and for the comfort they provide, allowing the patient to use their orthosis continuously during the prescribed period for treatment and even for aesthetic reasons [2,3,4,5]. Until a few years ago, although technologically feasible, the production of customized orthoses required specialized know-how with very high costs and production times, which made the production of customized orthoses unfeasible on a large scale. Additionally, in many prostheses and orthoses applications, such as in the case of children whose rapid growth may dictate the need to produce a new orthosis after a short time, or in patients where changes in physiognomy can occur due to different stages of the disability or working conditions, the evolution of the pathology makes the adoption customized solutions impracticable, since they involve long waiting times (weeks or months) [4].

The adoption of additive manufacturing techniques, namely 3D-printing technologies, in the production of medical devices such as orthoses and prostheses includes the following benefits: (i) a reduction in costs and manufacturing times; (ii) increased dimensional accuracy and geometric adaptability; (iii) increased degree of personalization; (iv) topological optimization; (v) decrease in specialized labor; (vi) in situ fabrication; (vii) provision of ‘online’ services for the production of customized orthoses and prostheses; (viii) availability of these technologies in remote or needy regions; and (ix) multi-material incorporation. These benefits are seen as the main drivers of a new area of research in the health field [6,7,8,9,10].

Fused Deposition Modeling (FDM) and PolyJet manufacturing techniques are among the most used 3D-printing technologies, the first being one of the most economically versatile and accessible methods. The range of materials available for additive manufacturing has been expanding significantly to respond efficiently to the need to provide personalized, sustained, simplified, and quick-way orthoses and prostheses with reduced weight and thickness without loss of features such as structural resistance, comfort, and flexibility [11,12,13]. However, the mechanical, chemical, and physical properties of materials such as biocompatibility (hypoallergenic), durability, susceptibility to external agents (humidity, ultraviolet radiation, biological fluids, cleaning agents, etc.), thermal and electrical characteristics, resistance to voltage loads and flexion, stiffness and elasticity, ease of cleaning/hygiene/sterilization, and ease of processing should be considered [14]. Moreover, the cost of materials and the selection of printing technology should not be neglected, as they determine the economic sustainability of the production of customized medical devices.

There is a need to use more than one material or a combination of several materials in certain types of orthoses and prostheses, which are normally the more temporary and permanent and more complex and dynamic ones, e.g., leather, metals, elastomers (such as rubbers, silicone, or foams), polymers, and ceramics. Further, despite their higher costs, due to their high stiffness and the possibility of obtaining structures with anisotropic properties that characterize them, carbon fiber composites are often used in these types of applications [15,16,17].

The flexibility offered by additive manufacturing opens the possibility to produce structures with spatial variation of mechanical, chemical, or other properties at the macroscopic level, i.e., to produce structures with functional gradients through the possibility of multi-material printing of two or more types of materials simultaneously [18]. Several studies point to the interest in multi-material manufacturing solutions as a way to include particular characteristics such as controlled elasticity. Among the most used materials are Polyamide (PA), Acrylonitrile Butadiene Styrene (ABS), Polycarbonate (PC), and Polypropylene (PP) due to their excellent properties, such as low density, low cost, flexibility, and ease of processing [2,4,15,19,20].

Given that the properties of the processed material are highly dependent on the processing parameters, namely the material’s printing direction, morphological studies (Micro-CT), mechanical tests (compression, traction, and fatigue), and chemical and thermal analyses (FTIR, DSC, and TGA) that report the influence of processing parameters on the mechanical, chemical, and thermal properties of these polymers are easily found in the literature. However, it is rarer to find published results on the impact strength of the above-mentioned materials printed by additive manufacturing, which is crucial in the case of certain orthosis applications [21]. It is also important to know the resistance of polymeric materials when in contact with biological fluids such as human sweat. As such, this paper aims to study the impact strength of nine polymeric materials manufactured by FDM. Additionally, the impact strength of these materials when immersed in artificial sweat for different periods is evaluated. Fracture surface analyses and water contact angle measurements were also conducted. To obtain a deeper understanding of the performance, durability, and suitability of these polymeric materials, studying the mechanical behavior and failure mechanisms is fundamental. The findings of this experimental research will contribute to the development of more effective and reliable orthotics using additively manufactured polymeric materials, leading to the improvement of patient outcomes and quality of life [22].

In more detail, the present paper aims to achieve three key research objectives:

- It is important to understand sweat’s influence on polymeric materials’ mechanical properties. The materials were subjected to artificial sweat with a pH of 6.3, simulating physiological conditions. By analyzing the changes in mechanical behavior resulting from exposure to sweat, the present study aimed to gain insights into the suitability of these materials for applications involving contact with human skin;
- The impact strength of the samples was evaluated in different printing orientations (vertical and horizontal) and under varying environmental conditions, including exposure to air and immersion in artificial sweat. The purpose of conducting these tests was to determine the materials’ ability to withstand impact forces and identify any variations in their performance based on different orientations (i.e., determining which orientation results in optimal mechanical properties and performance) and environmental exposures. This information can help select the most suitable material and orientation for the specific application;
- The use of additively manufactured polymeric materials for medical orthotics is attracting the interest of the scientific world nowadays; by understanding the mechanical behavior of these materials in different environmental conditions, including exposure to sweat, their suitability for orthotic applications can be determined and improved. This knowledge is crucial for developing orthotic devices that offer the necessary support and durability while maintaining comfort for the wearer.

## 2. Materials and Methods

The study was conducted on nine polymeric materials printed with 100% infill, considering an infill angle of 45° and a slice height of 254 μm. These materials are ABS (Acrylonitrile Butadiene Styrene), NYLON 12, PC (Polycarbonate), PETG (Polyethylene Terephthalate Glycol), PLA (Polylactic Acid), TPU (Thermoplastic Polyurethane), PC ABS (Polycarbonate with Acrylonitrile Butadiene Styrene), and high-performance polytherimide (PEI) thermoplastics ULTEM™ 1010 and ULTEM™ 9085. All materials were supplied by Stratasys (Stratasys, Eden Prairie, MN, USA). Some reference properties taken from the supplier’s material data sheet are summarized in Table 1. A Stratasys F170 printer (Stratasys, Eden Prairie, MN, USA) was used to print the specimens.

Three conditions were studied: dry environment, 24 days of immersion, and 189 days of immersion. Two different printing orientations were used for each type of material: vertical (V) and horizontal (H). These samples were printed in a prismatic geometry, precisely measuring 90 × 10 × 4 mm^3^. Each case was repeated three times (n = 3), resulting in a total of 54 specimens tested for each condition.

For the immersion process, an artificial sweat was used with a pH of 6.3. This artificial sweat solution is called phosphate-buffered saline [23,24]. To control mass change and the liquid absorption percentage of each sample after immersion, the width, thickness, and mass of samples were measured before each test; the mass was measured 3 times for the specimens immersed for 24 days and 5 times for the specimens immersed for 189 days. During the immersion time, the specimens were maintained at room temperature (20–24 °C). The mass was measured by an analytical balance scale accurate to a tenth of a milligram, model Phoenix GH-202 (A&D Company, Toshima City, Tokyo, Japan).

The wettability of the different materials was measured using the sessile drop method with the Theta Lite optical tensiometer equipment (Attension, Biolin Scientific, Gothenburg, Sweden), in which the contact angle was measured by OneAttension 1.0 software (Biolin Scientific, Sweden) right after the water droplet came into contact with the material. The values are expressed in degrees (°), and then the average and the standard deviation (SD) were calculated (n = 3).

For the impact tests, a Charpy Pendulum Impact testing machine with a 5 J hammer was used, model Instron CEAST 9050 (Instron, Norwood, MA, USA). These tests were performed following the recommendations outlined in the ISO 179 standard [25]. The Charpy impact test is a high strain rate test with a controlled weight pendulum swung from a set height. Each test was repeated three times (n = 3). The impact test helps to measure the amount of energy absorbed by the specimen during fracture. The absorbed energy was accounted for in percentage (%) of Joule. It is obtained based on the difference between the initial potential energy of the hammer and the residual energy after the impact. The software associated with the equipment directly gives this value.

The fracture surfaces of each specimen were captured using optical microscopy. Optical microscopy or light microscopy uses visible light and a system of lenses to enlarge images of small objects. The fracture surface analyses were carried out by visual analysis of the images. This evaluation aimed to identify failure mechanisms and assess how different environmental conditions affected the mechanical behavior of polymeric materials.

Statistical differences between each condition were evaluated on GraphPad Prism 10 software (GraphPad Software, La Jolla, CA, USA) with multiple unpaired *t*-tests. All tests were calculated with a confidence interval of 95%, where statistically significant differences are represented by *p* < 0.05, *p* < 0.01 and *p* < 0.001.

## 3. Results and Discussion

**Figure 1:**
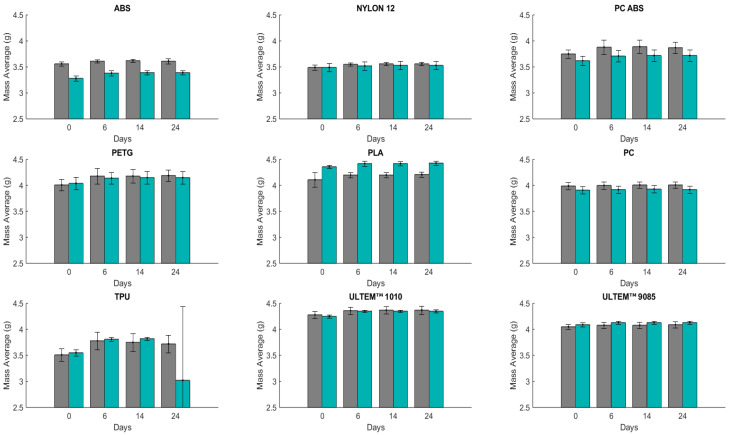
Mass average (mean ± SD) for different materials (H in gray and V in green) upon immersion in artificial sweat for 24 days (n = 3): ABS; Nylon 12; PC ABS; PETG; PLA; PC; TPU; ULTEM™ 1010; ULTEM™ 9085. The results of mass average for ABS, NYLON 12, PC ABS, and PETG were taken from Reference [21].

**Figure 2:**
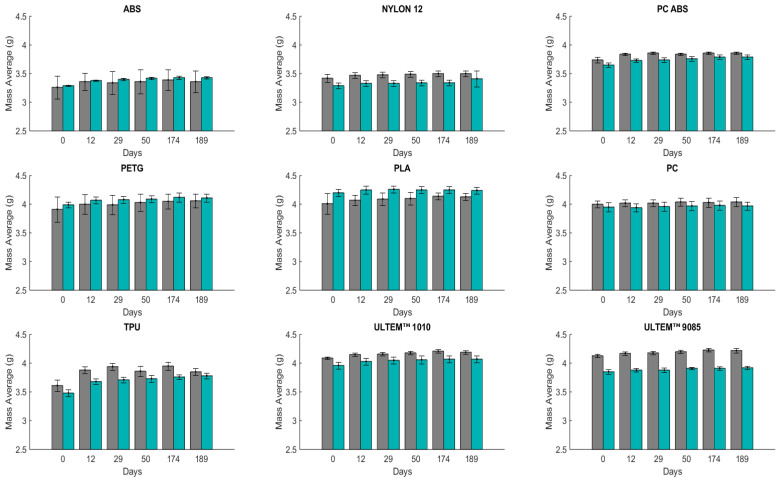
Mass average (mean ± SD) for different materials (H in gray and V in green) upon immersion in artificial sweat for 189 days (n = 3): ABS; Nylon 12; PC ABS; PETG; PLA; PC; TPU; ULTEM™ 1010; ULTEM™ 9085.

Submerging specimens in artificial sweat helps to determine its effect on the material’s mechanical properties for short-term (24 days) and long-term contact (189 days). Figure 1 and Figure 2 show the results in terms of mass change over time for the two manufacturing directions, (V) and (H). These values correspond to the average mass of the three samples.

The materials were conserved in an artificial sweat solution and weighed three times before the impact tests. The mass measurement for the first immersion of the polymers showed a distinct absorption percentage between the (H) and (V) printing orientation. Figure 1 presents the mass measurements for each interval period for 24 days of immersion.

In the first measurement, PC (H) with 0.31% of water absorption (WA) and PC (V) with 0.34% of WA showed lower liquid absorption, whereas TPU (H) with 7.51% and TPU (V) with 7.31% absorbed a greater amount. In the second measurement, TPU (H) with 6.77% and TPU (V) with 7.44% showed an increase in liquid absorption compared to the first one; meanwhile, PC (H) with 0.54% and PC (V) with 0.64% absorbed more liquid. In the third measurement, TPU (H) absorbed more liquid with 5.82%. However, it absorbed less than the previous two periods of immersion, and PC (H) with 0.57% and PC (V) with 0.36% absorbed less liquid than other materials. Comparing the materials, PC is the least absorbent, and TPU is the highest.

All materials were submerged in artificial sweat solution for a more extended period (189 days) and weighed five times (Figure 2) before the impact tests. The same conclusion as in the 24 days of immersion can also be drawn from the mass measurements for 189 days of immersion of the polymeric materials. PC is still resistant, with the lowest liquid absorption percentage, and TPU is still absorbent, with the highest liquid absorption percentage. After 12 days, PC (H) has a WA of 0.62%, and a WA of 1.03% for the measurement performed after 189 days. For PC (V), after 189 days, the WA is 0.46%. Otherwise, TPU (H) has a WA of 7.28% after 12 days and 6.60% after 189 days, and TPU (V) has a WA of 5.71% after 12 days and 8.46% after 189 days.

After the immersion, all specimens were submitted to impact tests. The results of this assessment are presented in Table 2. The values displayed in the table are the mean absorbed energy derived from three tests executed for each respective material for (H) and (V).

Regarding the printing direction (H) and (V) for the dry environment, only PETG and TPU show statistically significant differences of *p* < 0.01, where (V) showed higher values of absorbed energy. In the wet environment (24 days), ULTEM™ 9085 shows a high statistically significant difference between the absorbed energy by (H) and (V), with a *p* <0.001. TPU shows again a *p* < 0.01 in its energy absorption. At 189 days, three materials show a statistical difference of *p* < 0.01.; these materials are ABS, PC ABS, and ULTEM™ 9085, e.g., ULTEM™ 9085 (V) absorbed 66.1± 6.7%, and ULTEM™ 9085 (H) absorbed 35.1 ± 2.6% of the energy released.

When comparing the materials over the immersion time, there were no statistical differences between the immersion times of 24 and 189 days (results not shown in Table 2). However, when compared to the dry condition, differences were found. For the (H) direction, ABS was always statistically different for 24 (*p* < 0.05) and 189 (*p* < 0.05) days, decreasing its values. For the (V) direction, the PC ABS material also decreased its absorbed energy value at 189 days (*p* < 0.01). PLA, on the other hand, led to an increase in the energy values at both 24 (*p* < 0.01) and 189 (*p* < 0.05) days. Finally, TPU also decreased the absorbed energy values at 24 days of immersion, becoming statistically significant for both types of printing (*p* < 0.05). Under a dry environment, NYLON 12 (H) and (V) showed the highest absorption percentage, with 67.90± 3.00% and 64.50 ± 0.10%, respectively (~5% variance), indicating a good impact strength. PC was the material that presented the most similar results to NYLON 12, with a similar difference between the printing directions (~9% variance). The other materials presented the opposite behavior, with an improvement in the absorption percentage from (H) to (V) directions, and PETG presented the highest difference. Under wet environments, at 24 days, NYLON 12 (V) and PC (H) performed with 73.5 ± 20.5% (with ~14% variance from the (H)) and 71.7 ± 20.0% (with ~15% variance from the (V)), which are the highest energy absorption percentages, whereas PETG and PLA experienced a decrease in energy absorption in the (V) direction. The other materials experienced an improvement in energy absorption percentage in the (V) direction. At 189 days, PC (H) and ULTEM™ 9085 (V) performed with 77.5 ± 7.0% (with ~15% variance from the (V)) and 66.1 ± 6.7% (with ~88% variance from the (H)), showing the highest energy absorption percentage. An improvement in energy absorption can be seen in materials like ABS, PETG, PLA, TPU, and ULTEM™ 1010 for the (V). The remaining materials showed a decrease in energy absorption for the (V) direction. For all the conditions (wet and dry environments), TPU remained the material with the lowest energy absorption.

Comparing the (H) and (V) printing directions regarding the best-performing energy absorption materials, the tests performed in a dry environment and after 189 days of immersion for almost all materials exhibited the same trends for both (H) and (V), with differences in the absorbed energy percentage. Overall, samples printed in (V) absorbed more energy than samples printed in (H).

**Figure 3:**
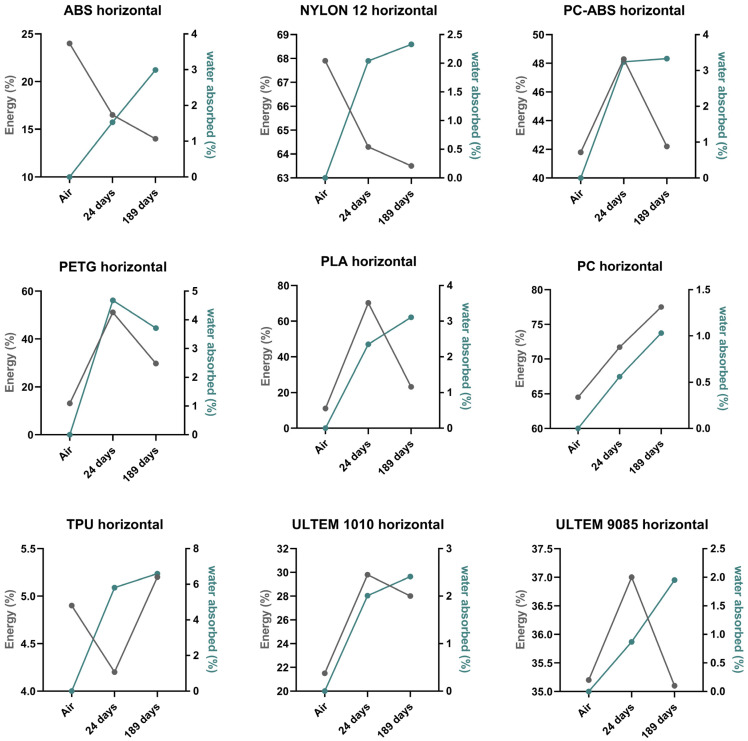
Comparison between absorbed energy (%) and water absorption (%) for horizontally printed materials.

**Figure 4:**
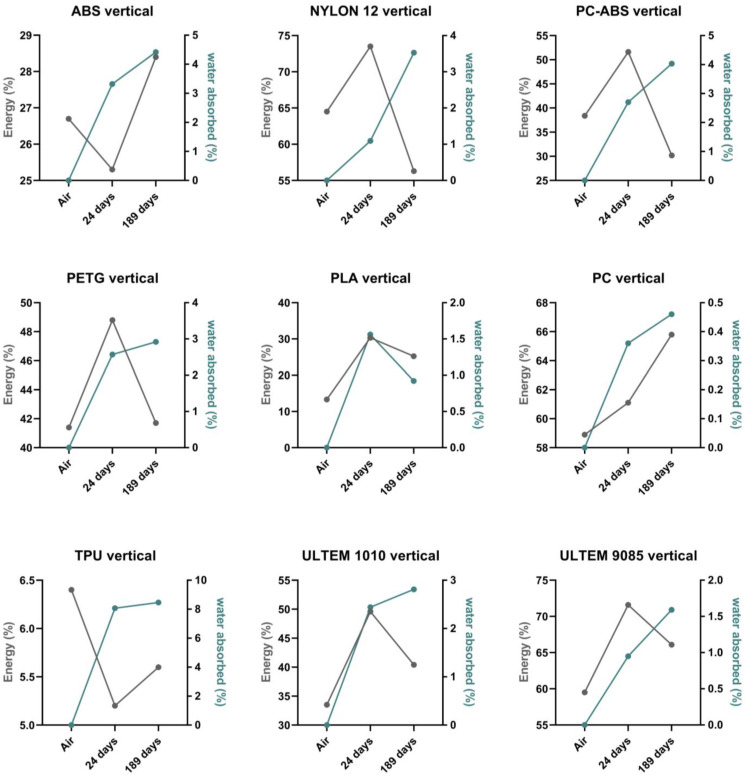
Comparison between absorbed energy (%) and water absorption (%) for vertically printed materials.

Figure 3 and Figure 4 show a comparison between absorbed energy and water absorbed of the nine polymeric materials for the different conditions (wet and dry environments) and printing directions (horizontal and vertical). For the sake of clarity, the y-axis scales differ from case to case.

Regarding the horizontally printed specimens (see Figure 3), it can be distinguished that ABS and NYLON 12 have the same energy absorption patterns, decreasing with an increase in WA over time. PC ABS, PETG, PLA, ULTEM™ 1010, and ULTEM™ 9085 also show the same energy absorption patterns, an increase in absorbed energy in 24 days and a decrease for 189 days, with a WA increase over time (for PC ABS, PLA, and ULTEM™ 1010) and a WA decrease at 189 days (PETG). PC energy absorption increased over time with increasing WA. Unlike PC, TPU’s energy absorption percentage decreased at 24 days and increased at 189 days, whereas WA increased over time.

Concerning the vertically printed specimens (see Figure 4), ABS and TPU exhibited a WA increase over time, and their energy absorption percentage decreased at 24 days and increased at 189 days. NYLON 12, PC ABS, and PETG had a WA increase over time, and the absorbed energy decreased at 189 days (more than the absorbed energy in the dry environment for NYLON 12 and PC ABS). PLA, ULTEM™ 1010, and ULTEM™ 9085 had the same patterns for absorbed energy. The WA for PLA decreased at 189 days. PC performed with an increase in both energy absorption and WA.

ULTEM™ 9085 showed different properties when tested in the (H) and (V) printing directions, as shown by the high percentage of energy absorption in samples printed vertically. This fact can be related to an anisotropic behavior, which refers to the properties of a material to give different results in different directions. ULTEM™ 9085 showed a high anisotropic behavior even for 189 days of immersion. There is a huge difference between the absorbed energy for specimens printed in the (H) and (V) direction, with 71.60% and 37.00% for the 24 days and 66.10% and 35.10% for the 189 days. Thus, it can be concluded that vertically printed ULTEM™ 9085 specimens exhibited the best energy absorption and the highest anisotropic behavior.

The results show that the choice of material and printing direction as well as the immersion time in artificial sweat significantly influence impact strength. Some materials, such as PC, NYLON 12, and ULTEM™ 9085, consistently performed well regardless of the immersion time, exhibiting low variations in energy absorption, whereas others experienced relevant changes in impact strength after immersion and may not be good for this type of application. Based on the experimental findings, regardless of whether the energy absorption is quite different, both Nylon 12 and TPU materials showed resistance to failure. Nylon 12 tended to bend rather than fail under the applied testing conditions. This suggests that Nylon 12 possesses excellent flexibility, allowing it to withstand high deformation. As the results show, PC may be more suitable for long-term applications, and NYLON™ 12 for short-term applications, in contact with sweat where impact strength is an important factor.

The impact strength results, both before and after immersion, alongside the mass measurement, provide deep insights into the performance of the polymeric materials manufactured by 3D printing under specific conditions (dry and wet environments). This can help in the material selection for biomedical applications in which orthotics are in contact with the human body, sweat, and the environment and in which orthotics may face different forces.

**Figure 5:**
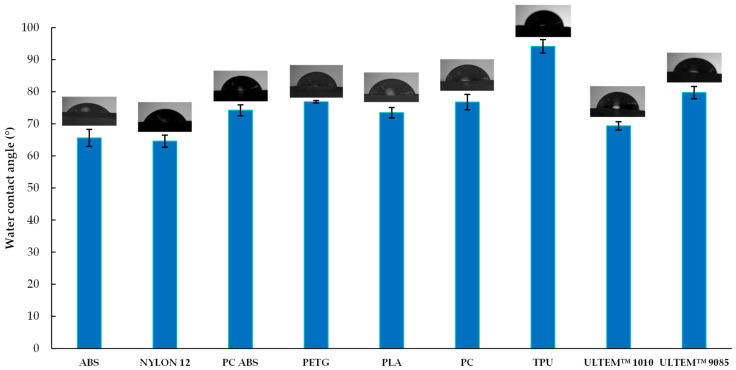
Wettability behavior (n = 3) of different polymeric materials: ABS; NYLON 12; PC ABS; PETG; PLA; PC; TPU; ULTEM™ 1010; and ULTEM™ 9085.

The water contact angle test can help to provide deeper knowledge of the hydrophilic and hydrophobic properties of the materials. Figure 5 summarizes the wettability behavior of different polymeric materials. TPU shows hydrophobic properties, since it obtained a water contact angle greater than 90°. However, it was the material that absorbed the most water (Figure 3 and Figure 4). TPU is a hygroscopic material [26], meaning that it can absorb moisture, a property already reported in the literature [27,28]. This highlights a clear distinction between surface properties (contact angle) and bulk properties (water absorption). Moreover, it may also have a porous structure (microscopic pores). Although not affecting the initial water droplet, this enables gradual liquid absorption over time. Thus, the water contact angle shows the initial interaction with water, and immersion tests reveal how the material behaves over time.

All other materials are hydrophilic, as they have contact angle values between 64° and 79° (Figure 5). A low contact angle value is associated with high surface energy [29,30,31]. Therefore, when comparing the contact angle results with those of the absorbed energy of NYLON 12, it is possible to observe that this material has the lowest contact angle and one of the highest absorbed energies. For TPU, it is possible to obtain the same correlation, because this material presented a higher contact angle and a much lower absorbed energy than the other materials (see Table 2). Therefore, it can be concluded that the hydrophobicity or hydrophilicity properties of the materials impact the mechanical properties of the material. Figure 5 also illustrates the standard deviations across all materials tested. The values determined exhibited some discrepancies, varying from 0.389 to 2.681.

**Figure 6:**
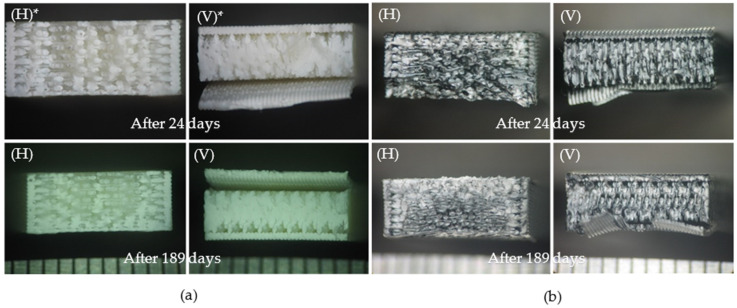

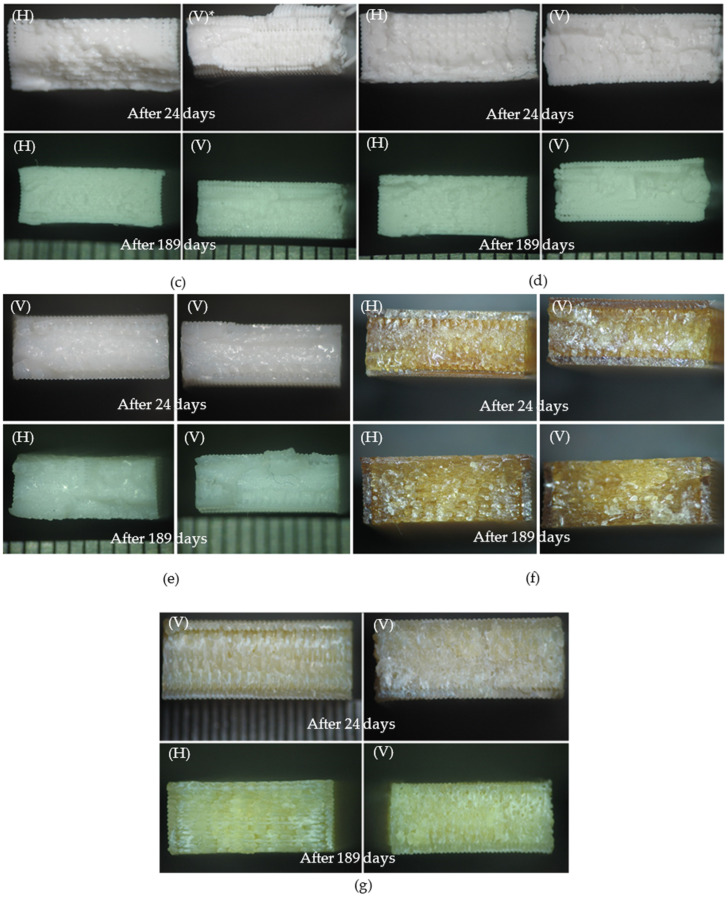
Fracture surface images of specific polymeric materials: (**a**) ABS; (**b**) PC ABS; (**c**) PETG; (**d**) PLA; (**e**) PC; (**f**) ULTEM™ 1010; and (**g**) ULTEM™ 9085. Images identified with * were taken from Reference [21].

In the world of 3D-printing manufacturing, a microscopic examination of material failures offers invaluable insights into the fracture behavior of the materials. Specimens printed using FDM at a high speed can absorb more energy before failure compared to others [32]. In this study, nine polymeric materials were studied. As discussed above, some of the material’s ability to absorb energy decreased after immersion, but the fracture surface images do not show any differences in the structure of these materials before and after the immersion, as can be seen in Figure 6. The artificial sweat into which the nine polymeric materials were plunged affected their resistance abilities but did not affect their microstructures. However, regarding NYLON 12, with good energy absorption, and TPU, with a high liquid absorption and low energy absorption, no conclusions can be drawn about their fracture surface characteristics, as these specimens did not fracture during the impact tests.

By analyzing materials’ fracture surfaces, the main failure mechanisms can be identified. Common causes were voids or pores and crack propagation, which resulted in ductile or brittle failure. Shear yielding mechanisms were also observed. ABS, for instance, may experience failure due to pores or spaces between layers (Figure 6a), resulting in brittle fractures and observable surface cracks. Crack propagation resulted in layer separation on the vertically printed ABS fractured surfaces. As shown in Figure 6b, PC ABS fracture surfaces display voids that hint at potential failure causes, highlighting the necessity for a comprehensive understanding of its brittle fracture behavior. The failure of PETG can primarily be attributed to crack propagation, likely facilitated by the presence of voids. Additionally, it exhibited a brittle fracture. The interior of the sample is melted, as can be seen in Figure 6c. The fracture surfaces of PLA (H) and PLA (V) show filament fusion and voids. There is crack initiation resulting in brittle fractures (Figure 6d). PC (H) and PC (V) are mostly melted inside, which could help the materials to withstand the forces applied. PC samples showed brittle fractures that may be caused by crack propagation (Figure 6e). ULTEM™ 1010 and ULTEM™ 9085, as shown in Figure 6f,g, revealed crack propagation and pores due to entrapped gas or due to lack of fusion. ULTEM™ 9085 (V) fracture surfaces show more voids. These showcase the distinct nature of polymeric material failure and accentuate the importance of microscopic analysis in 3D-printing materials, which can help to better determine the failure mechanisms.

## 4. Conclusions

This study addresses the impact strength of nine polymeric materials printed by additive manufacturing and immersed in artificial sweat solution. The analysis comprised three different immersion times (0 days, 24 days, and 189 days) and two printing directions (horizontal and vertical). Wettability and failure mechanisms were also examined. The following conclusions can be drawn:

- ULTEM^TM^ 9085 samples showed an anisotropy behavior during the impact test. This reveals an important consideration in the choice of printing orientation.
- Vertically printed samples displayed higher energy absorption than those printed horizontally. Materials like PC and NYLON 12 consistently displayed strong impact strength.
- For a long-term use, PC would be a good choice as a material for the manufacturing of orthotics because of its resistance to liquid penetration and its good energy absorption.
- For a short-term use, NYLON 12 would be recommended because of its high energy absorption percentage after 24 days of immersion.
- Failure was associated with the presence of voids or pores along with crack propagation. PC, which exhibited the greatest energy absorption, showed a melted fracture surface.

Conducting impact tests on these polymeric materials and studying their mass variations under specific conditions and their wettability helped to assess their resilience to impact forces and their reaction to sweat and water.

## Figures and Tables

**Table 1:** Reference properties of the tested polymer materials. Values taken from the supplier’s material data sheet.

Material	Filament Diameter (mm)	Ultimate Tensile Strength (MPa)	Elongation at Break (%)
ABS	1.75	28.1	8.1
NYLON 12	1.75	49.3	6.1
PC ABS	1.75	36.5	3.0
PETG	1.75	29	140
PLA	1.75	65.5	4.3
PC	1.75	57.9	4.9
TPU	1.75	15.6	552
ULTEM™ 1010	1.75	79.2	4.0
ULTEM™ 9085	1.75	68.1	5.4

**Table 2:** The average absorbed energy in dry and wet environments (in percentage with specific standard deviation) of 9 polymeric materials according to different printing conditions, (H) and (V) direction (n = 3). The highest and lowest values found for each condition are highlighted in bold. Statistical analysis was carried out using multiple unpaired *t*-tests, with differences represented by ** *p* < 0.01 and *** *p* < 0.001 when comparing the horizontal with the vertical printing, and differences represented by # *p* < 0.05 and ## *p* < 0.01 when compared to the dry environment and within the same type of printing, since no statistical differences were found between the 24-day and 189-day conditions.

Materials	Dry Environment (%)				Wet Environment (%)				
					After 24 Days		After 189 Days		
	(H)	(V)	*p*-Value	(H)	(V)	*p*-Value	(H)	(V)	*p*-Value
ABS	24.0 ± 2.2	26.7 ± 1.7	NS	16.5 ± 1.0	25.3 ± 3.3	NS	14.0 ± 3.8	28.4 ± 3.1	**
	-	-	-	#	NS	-	#	NS	-
NYLON 12	**67.9 ± 3.0**	**64.5 ± 0.1**	NS	64.3 ± 1.4	**73.5 ± 20.5**	NS	63.5 ± 3.7	56.3 ± 3.8	NS
	-	-	-	NS	NS	-	NS	NS	-
PC ABS	41.8 ± 15.5	38.4 ± 1.6	NS	48.3 ± 3.6	51.6 ± 16.0	NS	42.2 ± 2.3	30.2 ± 1.2	**
	-	-	-	NS	NS	-	NS	##	-
PETG	13.1 ± 6.3	41.4 ± 3.6	**	51.1 ± 35.0	48.8 ± 21.1	NS	29.7 ± 10.5	41.7 ± 15.0	NS
	-	-	-	NS	NS	-	NS	NS	-
PLA	11.1 ± 0.6	13.3 ± 3.2	NS	70.2 ± 40.2	30.3 ± 3.6	NS	23.2 ± 9.0	25.2 ± 5.0	NS
	-	-	-	NS	##	-	NS	#	-
PC	64.5 ± 4.7	58.9 ± 4.3	NS	**71.7 ± 20.0**	61.1 ± 2.7	NS	**77.5 ± 7.0**	65.8 ± 1.2	NS
	-	-	-	NS	NS	-	NS	NS	-
TPU	**4.9 ± 0.2**	**6.4 ± 0.3**	**	**4.2 ± 0.3**	**5.2 ± 0.1**	**	**5.2 ± 0.6**	**5.6 ± 1.2**	NS
	-	-	-	#	#	-	NS	NS	-
ULTEM™ 1010	21.5 ± 2.1	33.5 ± 10.0	NS	29.8 ± 4.7	49.6 ± 9.6	NS	28.0 ± 3. 6	40.4 ± 11.0	NS
	-	-	-	NS	NS	-	NS	NS	-
ULTEM™ 9085	35.2 ± 3.5	59.5 ± 16.4	NS	37.0 ± 5.2	71.6 ± 1.8	***	35.1 ± 2.6	**66.1 ± 6.7**	**
	-	-	-	NS	NS	-	NS	NS	-

## Data Availability

Research details will be provided upon request due to legal reasons.
